# Riemannian Manifolds for Biological Imaging Applications Based on Unsupervised Learning

**DOI:** 10.3390/jimaging11040103

**Published:** 2025-03-29

**Authors:** Ilya Larin, Alexander Karabelsky

**Affiliations:** Center for Translational Medicine, Sirius University of Science and Technology, Federal Territory Sirius, 1 Olympic Ave., Sirius 354340, Russia; karabelskiy.av@talantiuspeh.ru

**Keywords:** C2C12, t-SNE representation, Riemannian manifold

## Abstract

The development of neural networks has made the introduction of multimodal systems inevitable. Computer vision methods are still not widely used in biological research, despite their importance. It is time to recognize the significance of advances in feature extraction and real-time analysis of information from cells. Teacherless learning for the image clustering task is of great interest. In particular, the clustering of single cells is of great interest. This study will evaluate the feasibility of using latent representation and clustering of single cells in various applications in the fields of medicine and biotechnology. Of particular interest are embeddings, which relate to the morphological characterization of cells. Studies of C2C12 cells will reveal more about aspects of muscle differentiation by using neural networks. This work focuses on analyzing the applicability of the latent space to extract morphological features. Like many researchers in this field, we note that obtaining high-quality latent representations for phase-contrast or bright-field images opens new frontiers for creating large visual-language models. Graph structures are the main approaches to non-Euclidean manifolds. Graph-based segmentation has a long history, e.g., the normalized cuts algorithm treated segmentation as a graph partitioning problem—but only recently have such ideas merged with deep learning in an unsupervised manner. Recently, a number of works have shown the advantages of hyperbolic embeddings in vision tasks, including clustering and classification based on the Poincaré ball model. One area worth highlighting is unsupervised segmentation, which we believe is undervalued, particularly in the context of non-Euclidean spaces. In this approach, we aim to mark the beginning of our future work on integrating visual information and biological aspects of individual cells to multimodal space in comparative studies in vitro.

## 1. Introduction

In the past five years, progress has been made in tracking biological objects and semantic segmentation of cells and their components, including spatial orientation [1,2]. The detection and segmentation of nuclei have been considered the foundation of automated cellular image analysis. Arranging and understanding the morphological features of cells is a key factor in moving from qualitative to quantitative analysis. These methods support various quantitative studies, such as cellular morphology analysis, including size, shape, and other features. However, achieving reliable and accurate nucleus/cell segmentation remains challenging.

First of all, microscopic images contain background clutter with noise, artifacts (e.g., blurred regions), signal-to-noise ratio (SNR) limitations during image acquisition, and potentially poor contrast between the foreground and background. Furthermore, there are substantial variations in nucleus/cell size, shape, and intracellular heterogeneity. Nuclei/cells cluster or overlap due to differences in focal distance, partially obscuring each other. Complex pathological and microscopic images create challenges for manual analysis, leading to inter-laboratory discrepancies [3].

One advantage of automated methods is their ability to provide reproducible image feature measurements, which can be used in preclinical studies, enabling comparative research, prognosis development, and personalized medicine approaches [4]. The majority of studies are focused on the segmentation and tracking of biological objects. A study evaluating various U-Net architectures for nuclear and instance segmentation highlighted the importance of high-quality annotations [5]. Another work emphasized the need for isolating individual cells and defining cellular boundaries across tissues—a difficult task due to the close proximity of cells [6]. This direction is also critical for in vivo tasks, where higher cell density leads to reduced detection accuracy and boundary separation. This issue results in the over-segmentation of a single object. For example, artificial images and recursive waterflow post-processing have been recommended to address these challenges [7]. Thresholding approaches remain one of the most effective solutions for cell segmentation in terms of the Jaccard index [7]. In our previous work [8], we utilized the Data Science Bowl 2018 dataset, including 1500 prepared masks from complex fluorescent fibroblast images. Complex images were defined as those containing overlapping or clustered fibroblast cells. Post-processing involved threshold operations and morphological adjustments with a flexible kernel. The model initially detected nuclei, evaluated their parameters and image contrast, and iteratively adjusted the kernel size and elliptical structure. Data augmentation has proven effective in reducing overfitting, failures in F-score or IoU, and autofluorescence effects, particularly for fluorescent images across equal epochs. Geometric transformations and random erasures were the most effective techniques. A convolutional neural network (CNN) was applied to automate the recognition of C2C12 muscle cell differentiation based on phase–contrast images [9]. The group proposed a method to classify cells based on their morphological changes during differentiation, achieving a classification accuracy of 91.8%. A growing interest remains in using bright-field images to identify cellular morphological features.

Unsupervised morphological phenotyping approaches have also been employed to isolate and classify individual cells from low-resolution images [10]. Despite the potential of CNNs, these methods converge to fully convolutional networks (FCNs) and a Soft-max layer. While CNN-based methods successfully classify cells in mixed populations, they require extensive datasets and significant time for preparation compared to traditional staining or genotyping methods. A study by Bo Huang and colleagues predicted protein accumulation and localization within cells by analyzing protein sequences and amino acid compositions [11]. Machine learning algorithms trained on large datasets of labeled proteins revealed patterns and predicted subcellular locations. This approach enhances our understanding of cellular processes, facilitates drug discovery, and enables disease diagnostics. AINU (Artificial Intelligence for Nanoscale Nuclear Imaging), developed by Davide Carnevali et al., identifies cellular heterogeneity using nanoscale nuclear features [12]. This method demonstrates superior performance in distinguishing normal somatic cells, induced pluripotent stem cells (iPSCs), and cancer cells, outperforming traditional image analysis techniques. Another study introduced deep domain adversarial neural networks for the deconvolution of cell type mixtures in tissue proteome profiling [13]. This method effectively separated mixed signals from various cell types, providing more accurate proteomic profiles, which are critical for understanding cellular heterogeneity and disease mechanisms. State-of-the-art biomedical research has focused on morphological and cellular heterogeneity, driven by genetic changes, epigenetic modifications, or other molecular factors [14]. Emerging technologies now allow the storage of critical information in embeddings, enabling the development of unified models with experimental evidence to identify novel correlations between healthy and diseased cells.

The authors [15] propose to extend the concept of Segment Anything (SA) to a non-Euclidean data space in the framework of Segment Non-Euclidean Anything (SNA). The graph-based approach involves dynamic recruitment of neurons according to feature dimensionality, translating NLP/SA prompt to the graph. In this case, the approaches contribute to the movement in Graph General Intelligence. Xiaoyu Liu et al. [16] proposed a two-stage solution: U-Net extracts embeddings and edge map, on which superpixel segmentation is performed, and then a graph network based on Edge GNN combines the superpixels into classes—CNN + GNN on AC3/AC4 datasets.

The directions of integration of topological constraints and geometric invariants into the segmentation process remain relevant. In Shanru Lin et al. [17], the TopoUT method is proposed, introducing special regulators: loop penalty and cohesion penalty. These terms evaluate the 0th and 1st order topological characteristics (connectivity components and loops) and encourage the network to form correct cell boundaries, despite the importance of Betti error or splits/merges (IoS/IoU) score and the applicability of boundary loss [18] segmentation of curvilinear structures with a supervised/semi-supervised network an open problem.

**Figure 1:**
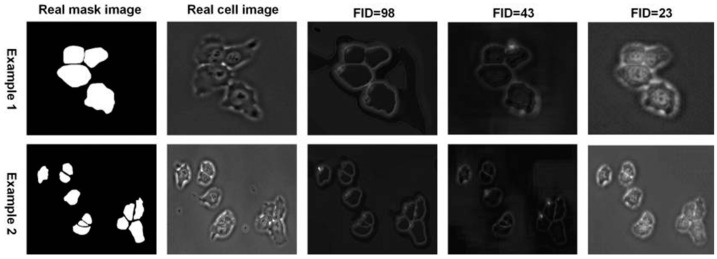
Impact of style injection and VGG perceptual loss on cGAN-Seg image generation. Two examples of comparing the effect of the style injecting technique and VGG perceptual feature loss function on cGAN-Seg performance to generate images of DeepSeas embryonic stem cells.

Unsupervised segmentation has seen rapid development in an effort to reduce reliance on time-consuming labelling of biomedical images. Autoencoders and self-supervised networks, clustering methods, and generative models for segmentation have emerged as several key strategies. The hallmark of all these approaches is the use of hidden data patterns, synthetic transformations, or working with gradients in layers instead of explicit class labels. AD-GAN [19] integrates the generation and segmentation processes into a single model. This network decorrelates the representation of the content (structure) and style (visual appearance) of a cellular image, achieving an image–mask transformation that preserves the geometry of the cells. AD-GAN outperformed other featureless methods by an average of 17.8% on Dice loss, and on the Cell Tracking Challenge benchmark. The cGAN-Seg model [20] is based on CycleGAN and generates realistic phase–contrast and fluorescent cell images, which are then used as an additional training sample for any segmentation algorithm. At the same time, works for light-field, phase–contrast, or topological contrast images are rather scarce (Figure 1). The works presented are summarized in segmentation examples, but exclude a simple metric: IoU + SSIM metrics by area.

At the same time, we cannot talk about obtaining quantitative information, applicability, and validation of biophysical models of different modalities without extremely accurate delineation of cell regions. Approaches in object segmentation are not universal and require fine-tuning or selection of hyperparameters. Another problem that arises from this is that standard normalization techniques are not universal. Moreover, convolution filters in 2N Euclidean architectures do not take into account both the invariance of the object in the convolution depth and the rotational equivariance. Augmentation techniques do not remedy this to a first approximation. A spatial transformer allows the network to be made asymptotically invariant to rotations and scale changes by incorporating differentiable interpolation and rotation. Capsule networks proposed by Geoffrey Hinton encapsulate information about the state of a feature that is detected in vector form. The capsules encode the probability of detecting the feature as the length of the output vector. The state of the detected feature is encoded as the direction in which the vector points. In this work, we outline the limits of quantifying cell morphology as cell cycle stages by reducing a high-dimensional representation to a t-SNE representation. Finally, we highlight the Riemannian factorization method of covariance matrices for patches as an approach to structural representation in the statistical–geometric plane, which will allow us to approach the problem of unsigned segmentation in biological computer vision.

## 2. Materials and Methods

The C2C12 cell line was used in the study. C2C12 cells were cultured in complete DMEM medium (Paneco, Moscow, Russia) with high glucose containing 10% (vol) fetal bovine serum and 2 mM L-glutamine (Paneco, Moscow, Russia) at 37 °C, 5% CO_2_ and 85–95% humidity. When 60–75% confluence of C2C12 cells was reached on the surface of a 25 cm^2^ culture vessel, trypsinization was performed by adding 1.0 mL of 0.25% trypsin-EDTA solution (1:1), incubated for 3–5 min at 37 °C in an incubator, and microscopy was performed to determine the degree of trypsinization. After complete trypsinization, the cells were resuspended by adding 4.5 mL of the original medium. Subsequent experiments were performed 12 h after incubation by sectioning a total of 100,000 myoblast cells into six-well plates. Microscopic phase–contrast imaging was performed using a Selena Logos X microscope (Logos Bio, Anyang-si, Republic of Korea) equipped with an incubation chamber to provide optimal environmental control for live cell imaging.

The implementation utilizes frameworks such as PyTorch 2.6.0, which ensure high flexibility and computational efficiency on GPUs. In the experimental setup, the data are normalized, converted into tensor format, and divided into training and validation sets. Special care is taken to ensure the reproducibility of experiments by setting random number generators and limiting the number of threads. All computational analyses, including model training and data processing, were performed using NVIDIA A100 GPUs (NVIDIA Corporation, Santa Clara, CA, USA), which provided the necessary computational power for handling large datasets and training deep learning models efficiently.

We used the CellPose [21] gradient-based instance segmentation model and annotations prepared in CVAT.ai for t-SNE representation. We use an 80:20 split for training and validation. Individual cell images were cropped with bounding boxes and background masks for latent vector generation using models such as VAE, AE, AE with multi-head attention, and DDPM. The size of each individual cell is resized to 128 × 128 with normalization in grayscale channel mean and std equal to 0.5. At the same time, the original cell sizes and their corresponding masks were preserved separately. Each model extracted features with a depth of 256, kernel size of 4, stride of 2, and padding of 1. For VAE, the weight adjustment coefficient for KLD in the total loss function was set to −0.001. DDPM used sinusoidal time steps with a decaying frequency sequence 2500^(2i/embedding dimension)^ where 2500 was determined empirically as optimal. A scale range of frequencies (2500) leads to stable training and the best performance in that domain. The embedding dimension (sometimes called d_model_, or “time embedding dimension”) is simply how many features your sinusoidal/time embedding has in total. Gaussian noise with alpha steps and cosine scheduling was added to DDPM. MAE, SSIM, or LPIPS losses were used to train the encoder, latent layer, and decoder for all models. Key input parameters for the network included object position, cell background, and object boundaries. Tools like rotational invariance and padding masks were applied to reduce the influence of the first two parameters. For boundary training, Gaussian noise with a 3 × 3 kernel was added to all images.

## 3. Results and Discussion

### 3.1. Low-Dimension Representation

The use of latent representations allows for the accumulation of research object information, independent of time and location, providing a unified form of data recording. Modern methods have made initial strides in predicting the behavior of biological objects [22]. In addition to numerous attention-based methods, increasing focus is being placed on object physics [23]. For cell-based tasks, embedding layers serve as the foundation. High-quality latent representations of hidden features can be obtained using encoder or encoder–decoder models, such as convolutional networks, transformers, diffusion models, or Siamese networks. Most researchers in biological computer vision rely on architectures such as VGG, ResNet, or Vision Transformers (ViT), including sliding window variations, due to their high efficiency in extracting complex biological image features. These models are also applicable in multimodal approaches, such as joint embeddings like CLIP, which utilize cosine similarity and matrix diagonalization to combine information from different data sources for more accurate analysis or sampling.

**Figure 2:**
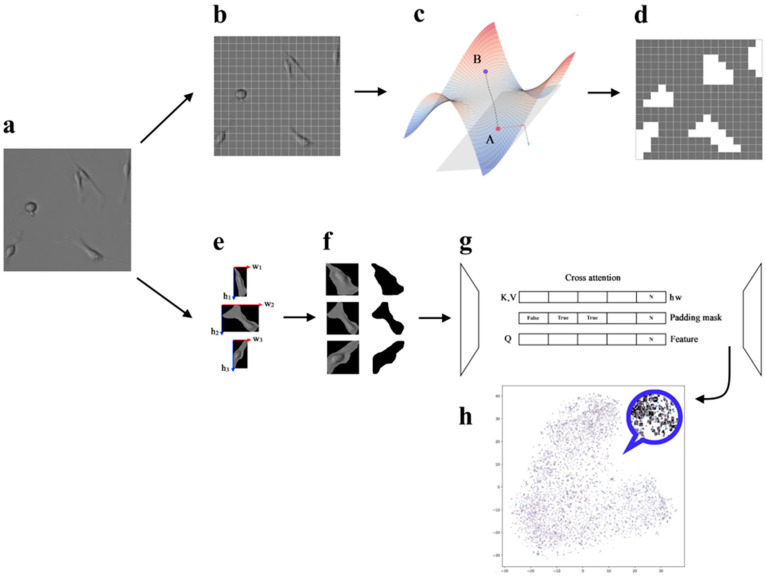
Flowcharts of t-SNE experiments and unsupervised segmentation based on the Riemannian manifold. Image (**a**), Splitting an image into patches (**b**), Translating the signs of patches into the Riemannian manifold (to illustrate Monkey Saddle (**c**), logarithm (red arrow), exponent (green arrow)), Classification of patches for the object (**d**); Cells in the bounding box (**e**) and the size (height—blue arrow, width—red arrow) for the shape vector, normalized images and padding mask (Boolean: true and false) acquisition (**f**), Encoder-decoder architecture with cross-attention (with padding mask) in the bottle neck between features and shape vector (**g**), t-SNE cell representation (**h**).

To achieve a high-quality latent space at the encoder output, multi-head attention with a Boolean mask is applicable (Figure 2f). The model was based on an autoencoder with cross-attention between the encoder output and the object’s shape (Figure 2e). The cross-attention layer was added to the bottleneck (Figure 2g). The additive union of the image features and shape vector was passed into it. The shape vector was obtained from MLP (2 linear layers, ReLU regularization and layer normalization). The learning rate was equal to 0.0001, the batch size was equal to 128 with ExponentialLR scheduler. Dimensionality reduction to 2 dimensions is performed using parametric t-SNE based on the Barnes–Hut method with a cosine similarity metric (Figure 2h). The use of cosine similarity is justified by the geometric invariance of cellular structures.

To enhance local adaptivity, the model processes image patches. Using unfolding and folding operations, the image is divided into patches and subsequently reconstructed into a spatial map. Additionally, clustering of the weights using the K-Means algorithm allows for the interpretation of the obtained coefficients as a probability distribution over classes (Figure 2d), which is demonstrated via visualizations of probability maps.

**Figure 3:**
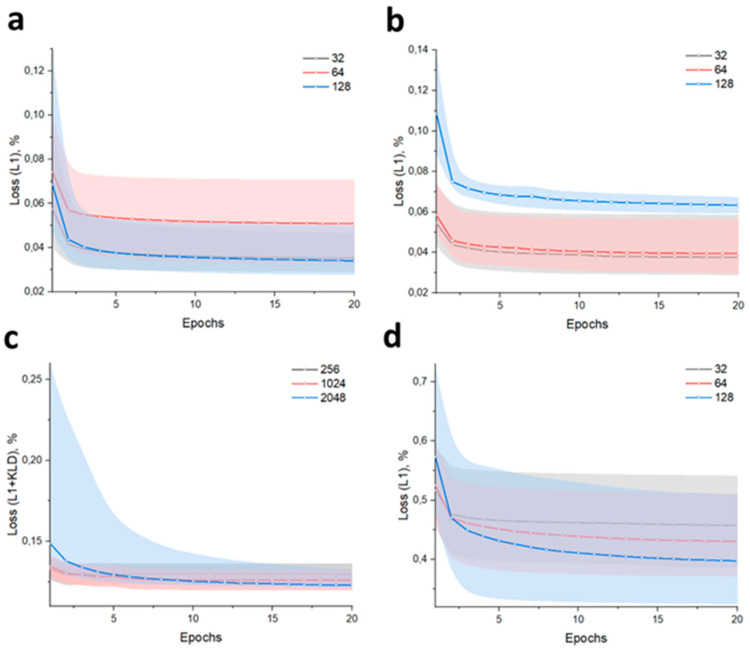
Model validation: AE (**a**), MHAAE (**b**), VAE (**c**), and DDPM (**d**). The borders of the corresponding color match a Sweeps W&B to automate hyperparameters search and visualize rich, interactive experiment tracking.

For data representation in two-dimensional t-SNE, we disregard the embedding-2 dimensions for simplicity. The value obtained from this representation corresponds to the passage within the experiment. Clustering in this dimensionality provides a metric that describes the growth rate of a given cell line at a specific passage under external factors, degeneration of the cell population, morphological relationships between passages within the experiment across different cell cycles, and much more. Network validation for reconstruction was also performed on the MNIST dataset. The dataset demonstrates artifacts of incorrect geometry due to the imperfect annotations generated by CellPose. The choice of architecture has a relatively minor impact in this case. However, in the VAE (Figure 3c), the training of a layer with a mean vector in a normal distribution allows for a smoother representation when dimensionality is reduced. For the latent layers of other architectures, the data remains interposable due to the high variability in cell morphology.

**Figure 4:**
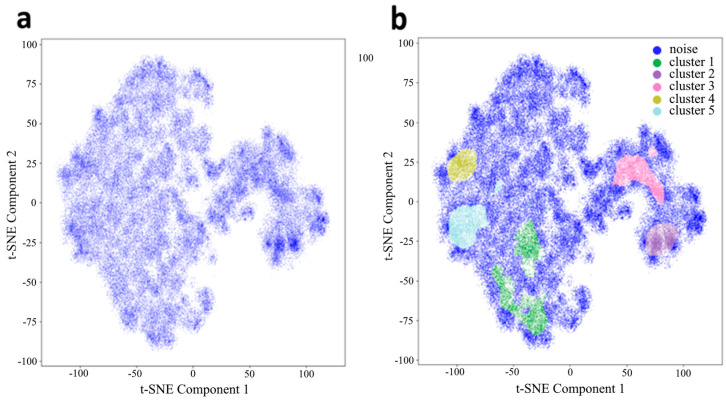
Low-dimensional representation of t-SNE of individual latent vectors from AE for cells (**a**) and cluster segmentation (**b**).

**Figure 5:**
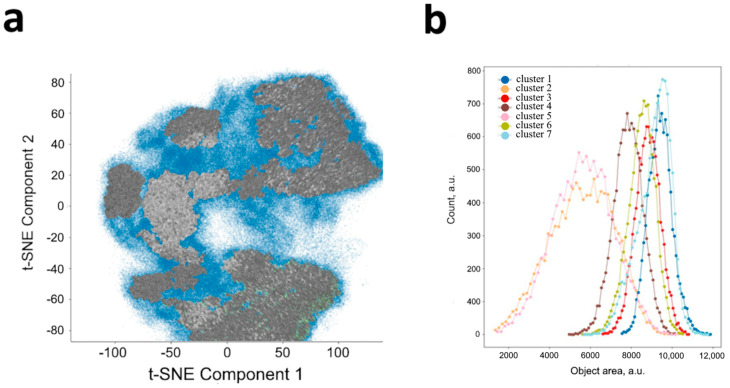
Image of cells in t-SNE representation (**a**), and the distribution of the shape of objects into clusters (**b**). An equally randomized number of elements was used for each cluster (SEM±: Cluster 1, 3, 4, 6, 7—4.5; Cluster 2, 5—8).

As shown in Figure 4a, when each individual cell is mapped, zones of increased density of the local data structure are formed. Each point represents a single cell. By performing clustering, for instance, using HDBSCAN, zones of high data density can be observed (Figure 4b). The map represents 100,000 points, and the density (alpha) is assumed to be 0.1 for better visualization. According to this, in the HDBSCAN method, the minimum cluster size was chosen 1–2% of the total cluster size, and the map shows 2%. We hypothesize that cells in different clusters correspond to different stages of the G cycle. When training the model on images of individual cells (with padding masks subtracted), it can be observed that the contribution of geometric variability to the formation of individual clusters is extremely high (Figure 5a). In contrast, the contribution of cell size (evaluated based on the bounding box of the object) is minimal (Figure 5b), as is the contribution of morphological heterogeneity. The latter is a factor that can be mitigated by combining size features with image features.

Let us build on the idea that molecular factors drive morphological changes. Accordingly, features should be extracted using encoder–decoder models. In this scenario, the key tools for analysis are the encoder output, decoder output, and the original image. Readers are correct in noting that mapping to latent space is neither a novel nor unique approach. With the advent of attention mechanisms, many methods have been adapted to efficiently incorporate these mechanisms into various architectures. Ultimately, everything depends on the creation of a high-quality dataset, specifically multimodal pairs.

This presents an evident contradiction when compared to the vast number of potential diseases. Furthermore, the question arises regarding the use of models with a “forgetting” mechanism, such as DiffKillR [24]. DiffeoInvariantNet is trained to ignore diffeomorphisms and geometric equivariant, while DiffeoMappingNet, on the contrary, is sensitive to diffeomorphisms and computes precise deformation fields between matched cells. The archetyping approach is a clear and straightforward method for clustering similar objects; however, the issue of cellular heterogeneity remains unresolved. Furthermore, when analyzing histological sections, each group may result in its own unique archetypes. The final encoder output was an additive combination of shape features extracted from cross-attention, image features, and raw image features. Within the bottleneck, the shape and image features were concatenated, followed by batch normalization and subsequent processing in the decoder. Additionally, the bottleneck underwent dimensionality reduction to 256 features using kernels 1 × 1, global pooling, flatten into a 1-dimensional, and L2 normalization.

**Figure 6:**
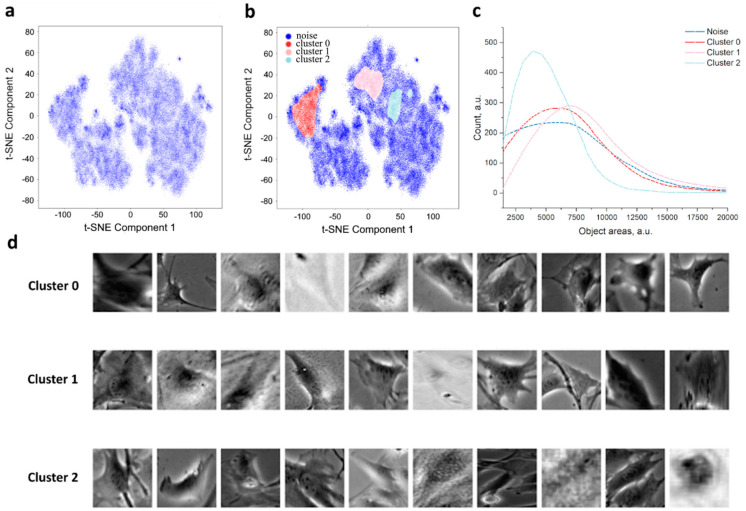
t-SNE representation of the data (**a**), clustering example (**b**), cell area histogram (**c**), and visualization of cells by clusters (**d**). An equal randomized number of elements was used for each cluster (SEM±: Noise—4.9, Cluster 0—11.6, Cluster 1—10.3, Cluster 2—7.2).

Let us make a logical assumption that our system contains at least two classes of objects and, according to the cell life cycle, four classes in total (Figure 6b). One of the obvious metrics is the cell area, specifically the area covered by an equal number of objects from each cluster. The objects were selected randomly. As seen in the two-dimensional representation, two large clusters are formed (Figure 6a). We will base our approach on the assumption that the chosen system contains two or more classes (Figure 6c). Other clusters may represent specific characteristics related to culture maintenance and/or experimental conditions (Figure 6d). Undoubtedly, the most critical aspect is the identification of cells. This is essential not only for tasks such as morphology analysis in unsupervised and semi-supervised segmentation but also for multimodal solutions.

### 3.2. Riemannian Manifold

Indeed, the inherent determinism of approaches to acquiring features from an object precludes the possibility of formulating a comprehensive list of the applicability of computer vision as a quantitative method. Numerous researchers have directed their efforts towards unifying the method. However, there has been a regrettable oversight in the research community of works on the classification of features in non-Euclidean space, taking into account cellular nature, form, and morphology.

We posed the question of whether it is possible to generate embeddings in an unsupervised manner and initialize joint representations with classes without data alignment, relying solely on their quantity. To explore this, we evaluated the feasibility of obtaining segmentations and embeddings using unsupervised learning based on Riemannian manifolds, positive definite matrices, and a dictionary built upon them. The approach described by Anoop Cherian and Suvrit Sra [25] served as the foundation for our method.

**Figure 7:**
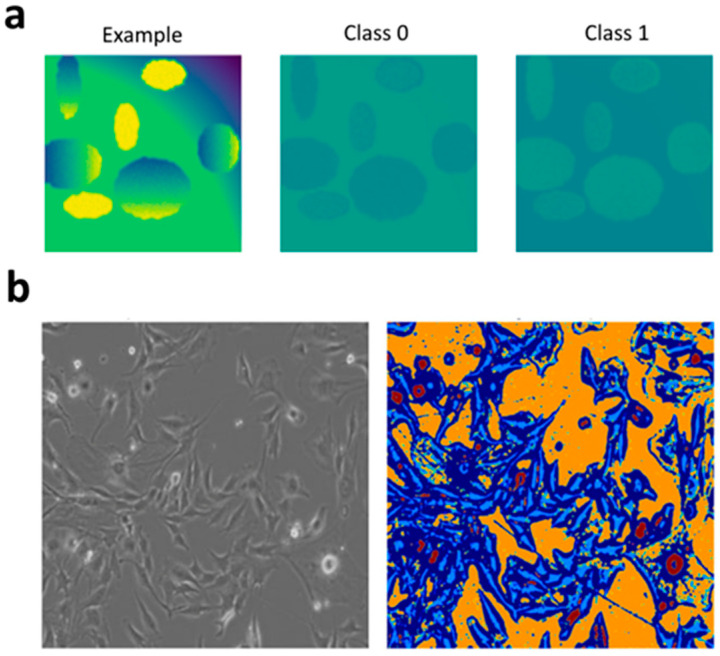
Probability map images for the test image (**a**), the original image ((**b**), left), and its probability map for 5 object classes ((**b**), right)).

In this work, we propose an architecture that combines deep learning methods with the processing of Symmetric Positive Definite (SPD) matrices within the framework of Riemannian geometry. This approach enables the extraction of informative features from images by computing covariance matrices, which are then encoded using a Riemannian Dictionary Learning model. Such a model is particularly relevant for segmentation and classification tasks, where taking into account the geometric structure of the data enhances the robustness and interpretability of the results. K-Means was employed for grouping the weights, with the construction of a probability map (Figure 7a) as a differentiable component. This approach demonstrates the feasibility of cell archetyping and generating high-resolution probability maps (Figure 7b) for cells under the microscope.

One of the key challenges when working with covariance matrices is to ensure their symmetry and positive definiteness. To address this, the following functions are implemented:

The function symmetrizes the input matrix and guarantees its positive definiteness by adjusting small eigenvalues. Consequently, the computed covariance matrices can be reliably interpreted as elements of the SPD Riemannian manifold.

(1) $$A_{SPD}=Q\underset{\epsilon \leq \lambda_{i}^{\prime }<\infty }{\max}\left(\Lambda ,\epsilon I\right)Q^{T},\;A=\frac{1}{2}(A+A^{T}),\;A=Q\Lambda Q^{T},\;\Lambda^{\prime }\leftarrow max\left(\Lambda ,\epsilon I\right)$$

To transition between the SPD manifold and its tangent space, logarithmic and exponential mappings are applied. In particular, the logarithmic mapping allows for the linear combination of matrices in the tangent space, while the exponential mapping returns the result to the manifold—a critical step for correctly constructing the dictionary atoms.

(2a) $$\log A=U\log\left(\Sigma \right)U^{T},\;\log\Sigma =diag(\log\sigma_{i}),\;e^{Y}=Vdiag\left(e^{\gamma_{I}}\right)V^{T}$$

$$Cov\left(X\right)=\frac{1}{hw-1}\left(X-\bar{X}\right){\left(X-\bar{X}\right)}^{T}+\epsilon I$$

$$A\overset{log}{\to }Z\overset{vec}{\to }z\overset{W}{\to }\omega \overset{\sum \omega_{k}Z_{k}}{\to }Z_{dict}\overset{mat}{\to }Y\overset{exp}{\to }\hat{A}$$

(2b) $$A=LL^{T}$$

The function of Stein divergence is used as the metric to measure the discrepancy between the original and the reconstructed matrix. This metric takes into account the Riemannian geometry of the SPD space and serves as the basis for the loss function.

(3) $$L=\frac{1}{N}\sum_{1}^{N}d_{Stein}\left(A_{i},{\hat{A}}_{i}\right)+\alpha {\left\|w\right\|}_{l_{1}}+\beta \sum {\left(\Delta w\right)}^{2},\;\nabla L\overset{update}{\to }\left(W,\left\{Z_{k}\right\}\right)$$

A CNN is used to extract local features from images. After partitioning the image into patches (Figure 2b), the data are centralized, and a covariance matrix is computed for each patch. To prevent singularity, a small regularizing epsilon matrix is added to the covariance matrix, which is then processed by the Function (1) to guarantee its positive definiteness. Covariance matrices are mapped to the tangent space using the Function (2a), which allows them to be represented in vector form. The vectorized representations are processed by a linear layer, yielding weights that characterize the contribution of each dictionary atom to the approximation of the original matrix. A linear combination of the dictionary atoms is performed in the tangent space, after which the Function (2a) maps the result back to the SPD manifold (e.g., the monkey saddle on Figure 2c). The Function (3) between the original and the reconstructed covariance matrices accounts for the Riemannian geometry. Instead of applying Function (2b), one can go the way of the Cholesky decomposition—such as adding a small multiple of the identity matrix or clamping small eigenvalues—to ensure that the matrix is SPD.

Given the considerations outlined above, we propose to operate directly within non-Euclidean spaces to develop novel methodologies and achieve new results for objects exhibiting equivariance, which we conceptualize as cells. This approach is motivated by the recognition that many objects of interest in fields such as computer vision and pattern recognition possess intrinsic geometric structures that are not adequately captured in conventional Euclidean frameworks. We leverage the natural geometry of the space to preserve the inherent symmetries and invariances of the data by working in a non-Euclidean (Riemannian) space. Specifically, our method utilizes logarithmic and exponential mappings to transition between the manifold of symmetric positive definite (SPD) matrices and its tangent space. The logarithmic mapping enables us to perform linear combinations in the tangent space—a crucial step for constructing a structured dictionary of “cells”—while the exponential mapping ensures that the results are correctly projected back onto the manifold. This two-step process is essential for maintaining the geometric fidelity of the representations.

## 4. Conclusions

In our framework, each equivariant object is treated as a cell, represented as an element on the manifold. By structuring these representations in a dictionary-learning context, we can effectively capture and model the complex relationships and interactions among the objects. This approach not only aligns with the theoretical principles of equivariance but also provides a powerful tool for enhancing the robustness and interpretability of models in practical applications. Ultimately, operating in these non-Euclidean spaces opens new avenues for the analysis and processing of geometrically complex data, leading to improved performance in tasks where traditional Euclidean methods may fall short.

Certainly, there is currently no universally accepted solution or pre-trained weights for comparing different models in the task of cell detection and classification. Unsupervised learning offers a potential for biological applications, enabling advancements in cell segmentation, clustering, and the analysis of cellular heterogeneity. In this study, we explored the use of latent representations and clustering to analyze individual cells and their morphological characteristics. We demonstrated that latent spaces can provide valuable insights into cell populations at different stages of the cell cycle. The use of techniques such as t-SNE for dimensionality reduction and clustering techniques revealed the potential for identifying subpopulations within cell cultures. These results contribute a new thesis to the hypothesis that morphological changes are closely linked to molecular factors, making latent inclusions a promising tool for understanding cell behavior and phenotype. Our results also underscore the importance of robust datasets and the integration of attention mechanisms to improve the quality of latent representations. Moreover, the application of Riemannian manifold learning and dictionary-based approaches holds promise for unsupervised segmentation and embedding generation without prior labels.

## Data Availability

The authors provide the data preprocessing code and the model development code on request.
